# Supplementary material for: Detection of magnetospheric ion drift patterns at Mars
Source: Nat Commun. 2023 Oct 27;14:6866. doi: 10.1038/s41467-023-42630-7 (PMC10611764; doi:10.1038/s41467-023-42630-7)
Supplement: Supplementary file 1 — Supplementary Information [file 41467_2023_42630_MOESM1_ESM.pdf]

Supplementary Information for

Detection of magnetospheric ion drift patterns at Mars

Chi Zhang[1,2,a], Hans Nilsson[3], Yusuke Ebihara[4], Masatoshi Yamauchi[3], Moa Persson[5], Zhaojin Rong[1,2,*], Jun Zhong[1,2], Chuanfei Dong[6], Yuxi Chen[6], Xuzhi Zhou[7], Yixin Sun[7], Yuki Harada[8], Jasper Halekas[9], Shaosui Xu[10], Yoshifumi Futaana[3], Zhen Shi[1,2], Chongjing Yuan[1,2], Xiaotong Yun[11], Song Fu[11], Jiawei Gao[1,2], Mats Holmström[3], Yong Wei[1,2,*], and Stas Barabash[3]

[1]Key Laboratory of Earth and Planetary Physics, Institute of Geology and Geophysics, Chinese Academy of Sciences, Beijing, China

[2]College of Earth and Planetary Sciences, University of Chinese Academy of Sciences, Beijing, China

[3]Swedish Institute of Space Physics, Kiruna, Sweden

[4]Research Institute for Sustainable Humanosphere, Kyoto University, Uji, Japan

[5]Graduate School of Frontier Sciences, The University of Tokyo, Kashiwa, Japan

[6]Center for Space Physics and Department of Astronomy, Boston University, Boston, MA, USA

[7]School of Earth and Space Sciences, Peking University, Beijing, China

[8]Department of Geophysics, Graduate School of Science, Kyoto University, Kyoto, Japan

[9]Department of Physics and Astronomy, University of Iowa, Iowa City, IA, USA

[10]Space Sciences Laboratory, University of California, Berkeley, Berkeley, CA, USA

[11]Department of Space Physics, School of Electronic Information, Wuhan University, Wuhan, China

[a]Present affiliation: Center for Space Physics and Department of Astronomy, Boston University, Boston, MA, USA

*Corresponding author: Zhaojin Rong (rongzhaojin@mail.iggcas.ac.cn)

Yong Wei (weiy@mail.iggcas.ac.cn)

This PDF file includes:

Supplementary Figures 1 to 6

Supplementary Table 1

Supplementary References

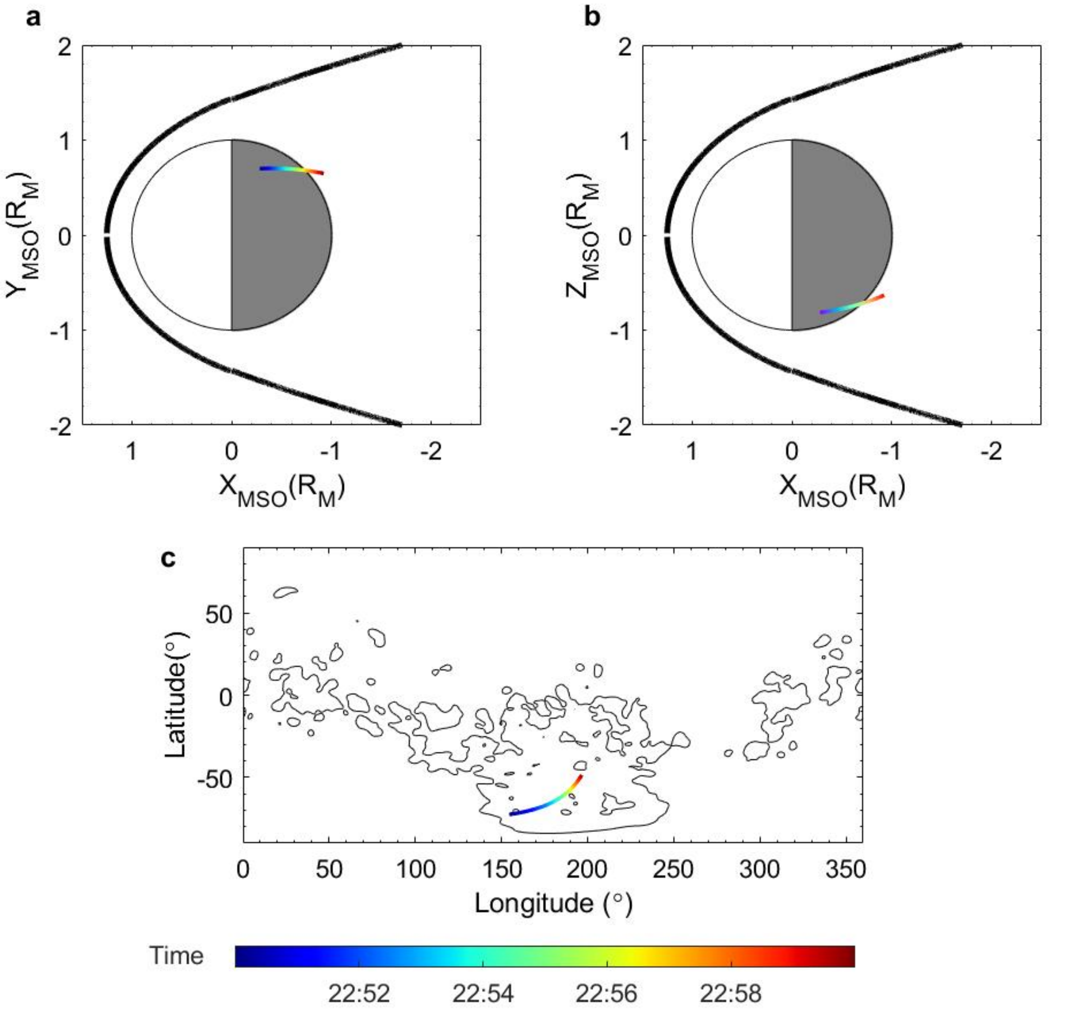

Supplementary Figure 1. **MAVEN's trajectory during 22:50-23:00, August, 2018**. (a), (b), and (c) show the trajectory of MAVEN in the $X_{MSO} - Y_{MSO}$, $X_{MSO} - Z_{MSO}$, and in Planetographic coordinates, respectively, during 22:50-23:00 UTC on April 15, 2018. The black dashed curves in (a), (b) denote the nominal magnetic pile-up boundary[1]. The black contour lines in (c) represents the crustal fields at an altitude of 180 km from the latest crustal fields model[2]. The colored curves represent the time along the trajectory of MAVEN. The Mars Solar Orbital (MSO) coordinates are defined as, the $\mathbf{X_{MSO}}$ points from Mars to the Sun, $\mathbf{Y_{MSO}}$ points opposite to the component of the orbital velocity perpendicular to $\mathbf{X_{MSO}}$, and $\mathbf{Z_{MSO}}$ completes the right-handed system. The Planetographic coordinates are defined as follow[3], the latitude represents the angular distance of a point on the planet's surface north or south of the equator. The latitude is measured from 0° at the equator to +90° at the north pole and -90° at the

south pole. The longitude of a point is defined as the angle between two halfplanes. The first half plane is the meridional plane that contains the point, and the second half plane is the meridional plane that contains the reference point for the prime meridian. The prime meridian is defined by the crater Airy-0. It is measured positively to the east from 0° to 360°.

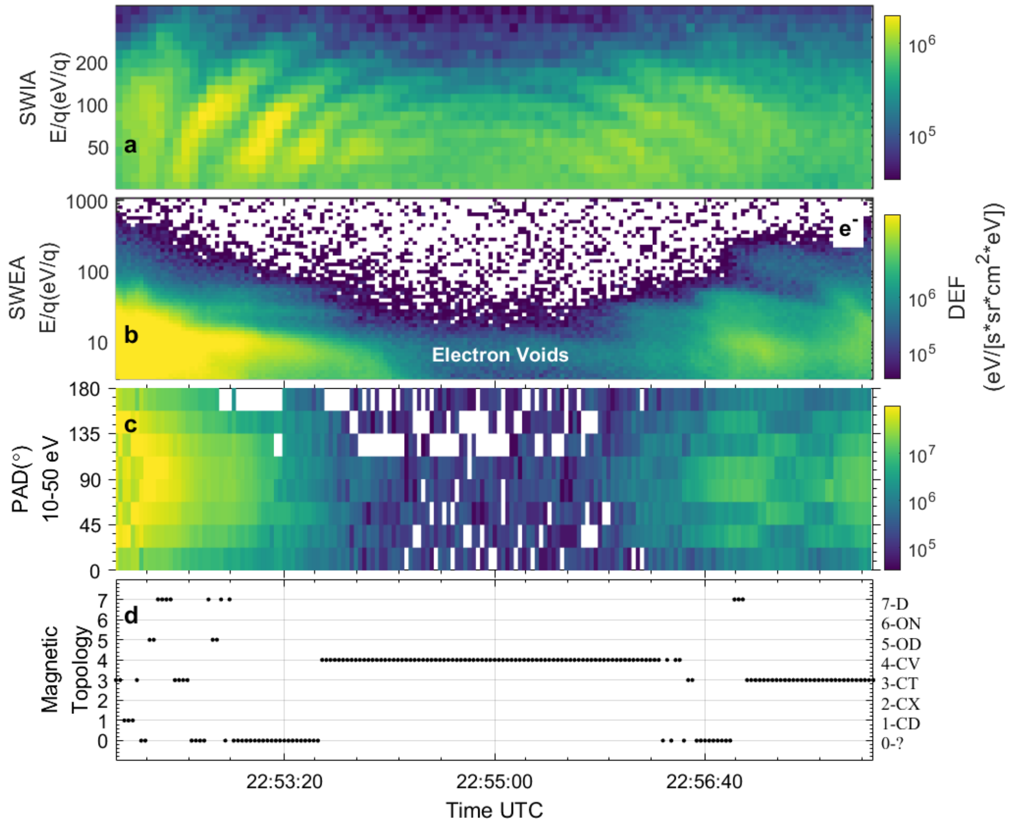

Supplementary Figure 2. Electron distributions and magnetic topology analysis. (a) Ion spectrum. (b) Electron spectrum observed by SWEA. (c) The pitch angle distribution of 10-50 eV electrons. (d) the magnetic topology index[4]. It can be seen that the electrons show a double-sided loss cone distribution, implying that the field lines were closed and connected to the ionosphere. The magnetic topology is categorized into seven types: (1) closed-to-day (C-D); (2) cross-terminator-closed (C-X); (3) closed-trapped (C-T); (4) closed-voids (C-V); (5) open-to-day (O-D); (6) open-to-night (O-N); (7) draped (DP). Therefore, (1), (2), (3), (4) typically represent the closed crustal fields. While (5), (6) represent the open field lines (OP). Apart from the unknown types, the magnetic field lines basically belong to the closed-voids (CV) and closed-trapped (C-T) types, which both are the closed crustal field lines with both foot-points located in the ionosphere.

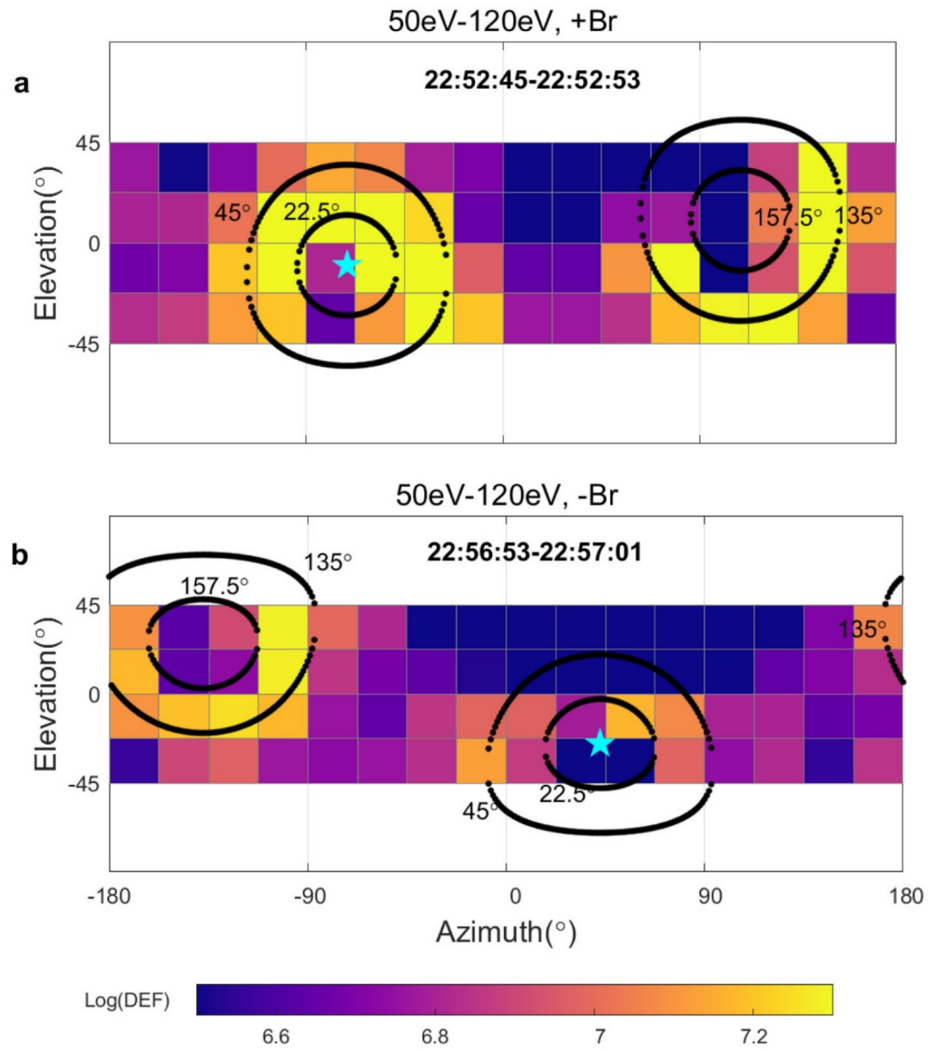

Supplementary Figure 3. SWIA observations of the angular distribution of the dispersed ions with energy ranging from 50-120 eV. (a) the observed angular distribution with positive Br during 22:52:45-22:52:53 UTC. (b) the observed angular distribution with negative Br during 22:52:45-22:52:53 UTC. The cyan pentagram represents the direction of the local magnetic field. The black circles represent the directions with a pitch angle of 22.5°, 45°, 135°, and 157.5°.

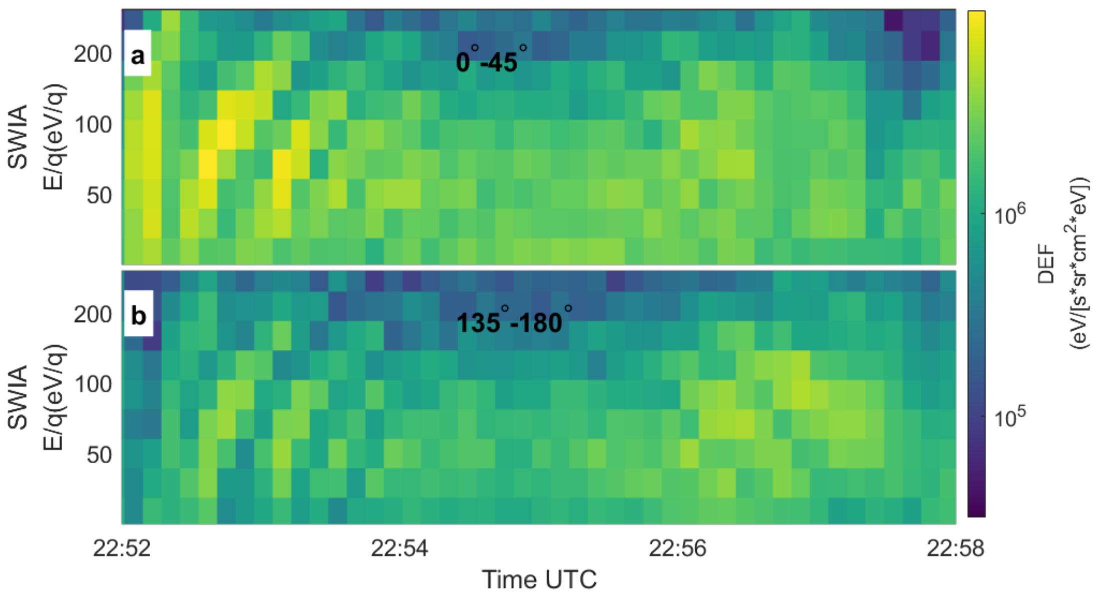

Supplementary Figure 4. SWIA observations of the differential energy flux of dispersed ions at 0-45° (upper panel) and 135°-180° (bottom panel) pitch angles. The dispersed structures occur simultaneously in both parallel and antiparallel moving directions, which contradicts the scenario of periodicity caused by repeated bounce motions.

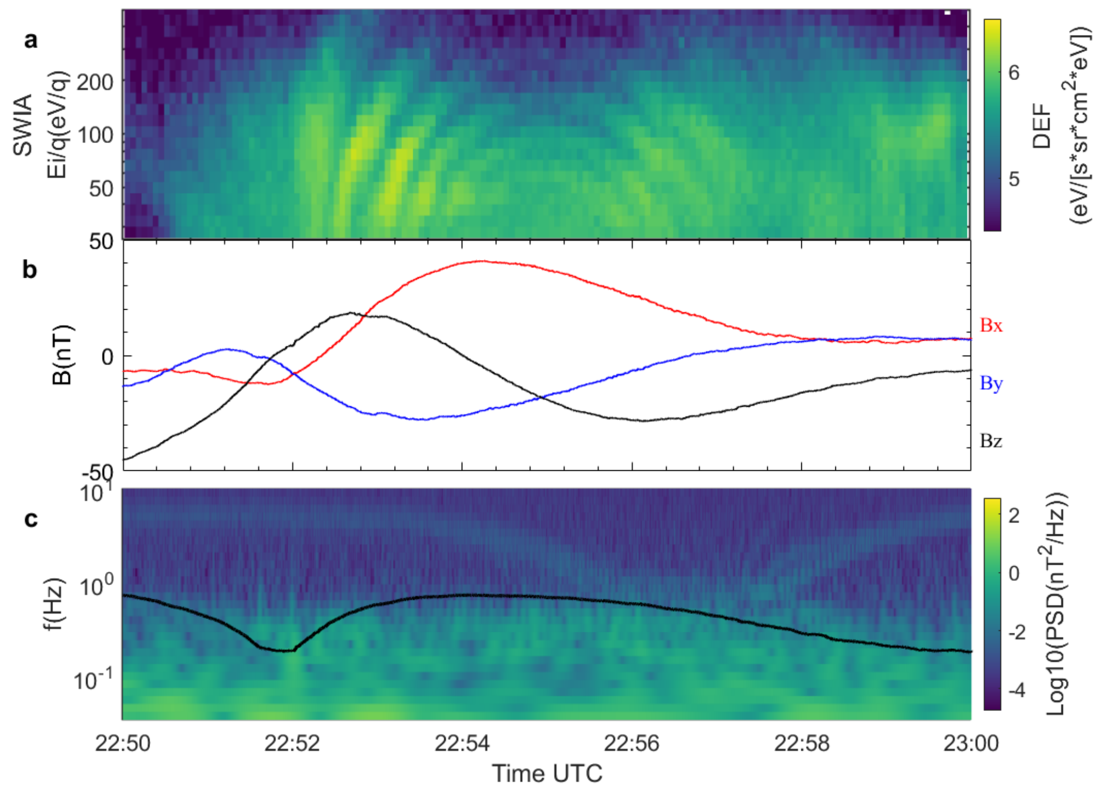

Supplementary Figure 5. Wave analysis. (a) Ion spectrum. (b) 32 Hz magnetic fields data. (c)The power spectral density (PSD) of the magnetic field. The black curve in c denotes the local gyro-frequency of protons. The y axis in (c) is the wave frequency. There are no obvious wave signatures during the observed event.

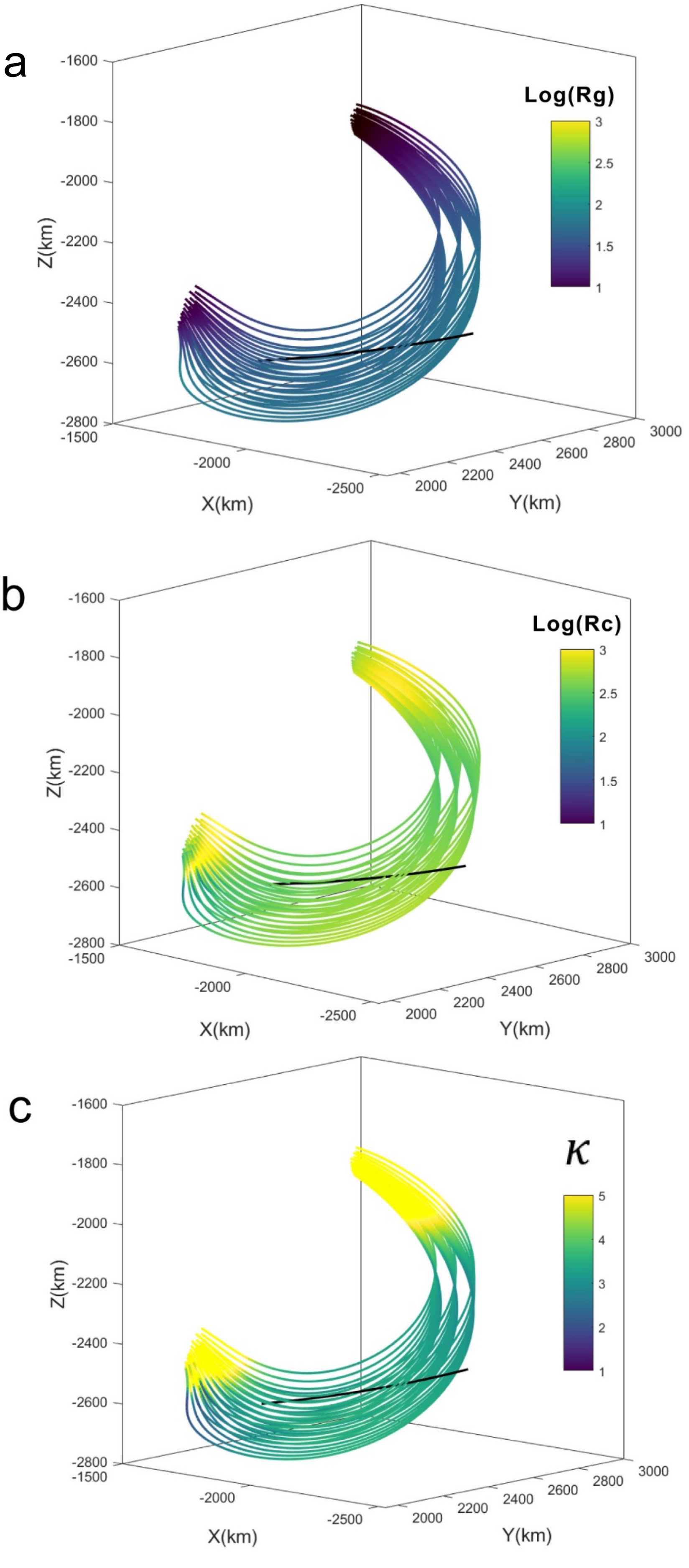

a
Log(Rg)
3
2.5
2
1.5
1
-1600
-1800
-2000
-2200
-2400
-2600
-2800
Z(km)
-1500
-2000
-2500
X(km)
2000
2200
2400
2600
2800
3000
Y(km)
b
Log(Rc)
3
2.5
2
1.5
1
-1600
-1800
-2000
-2200
-2400
-2600
-2800
Z(km)
-1500
-2000
-2500
X(km)
2000
2200
2400
2600
2800
3000
Y(km)
c
κ
5
4
3
2
1
-1600
-1800
-2000
-2200
-2400
-2600
-2800
Z(km)
-1500
-2000
-2500
X(km)
2000
2200
2400
2600
2800
3000
Y(km)

Supplementary Figure 6. The distribution of gyroradius, adiabatic parameter of 200 eV H^+ with pitch angle of 90°, and the curvature radius along the traced field lines. (a) the gyroradius of 200 eV H^+ with pitch angle of 90° (R_g). (b) the curvature radius ($|\mathbf{R_c}|$) of the field lines. (c) the adiabatic parameter, κ is defined as the square root of the ratio between the value of magnetic field curvature radius and the particle's gyroradius[5], that is $\kappa = \sqrt{|\mathbf{R_c}|/R_g}$, where $\mathbf{R_c}$ is the local curvature radius of field lines, that is $\mathbf{R_c} = \left((\mathbf{b} \cdot \nabla)\mathbf{b}\right)^{-1}$. R_g denotes the gyroradius of particles, that is $R_g = mV_{\perp}/Bq$. The particles basically adhere to an adiabatic motion for $\kappa \gg 1$, because the gyroradius of particles is negligible compared with the spatially variable scale of magnetic fields. The black curves in (a)-(c) are the spacecraft trajectory.

Supplementary Table 1. The list of the wedge-like dispersion events.

No	Time	Rising tone (Yes?)	Falling tone (Yes?)
1	2015-08-03 10:37:45-10:41:30	Y	Y
2	2015-12-05 08:24:00-08:31:00	Y	Y
3	2018-02-07 10:54:00-11:00:00	Y	Y
4	2018-02-25 17:38:30-17:41:00	Y	
5	2018-04-15 22:52:00-22:58:00	Y	Y
6	2018-05-06 00:51:50-00:54:30	Y	Y
7	2018-05-31 21:14:20-21:17:30	Y	Y
8	2018-07-03 12:34:37-12:35:52	Y	
9	2018-07-15 17:57:45-17:59:15	Y	
10	2018-07-17 18:49:15-18:56:00	Y	Y
11	2019-03-21 19:57:45-19:59:15	Y	
12	2019-03-23 21:22:00-21:24:00	Y	
13	2019-05-16 03:34:30-03:40:00	Y	Y
14	2019-06-02 01:27:00-01:29:30	Y	Y
15	2019-09-03 17:49:30-17:51:15	Y	
16	2020-04-29 21:27:00-21:29:00	Y	Y
17	2020-05-20 14:38:00-14:42:00	Y	

References

1. Trotignon, J. G., Mazelle, C., Bertucci, C., & Acuña, M. H. (2006). Martian shock and magnetic pile-up boundary positions and shapes determined from the Phobos 2 and Mars Global Surveyor data sets. Planetary and Space Science, 54(4), 357–369. https://doi.org/10.1016/j.pss.2006.01.003
2. Gao, J. W., Rong, Z. J., Lucy, K., Li, X. Z., Liu, D., & Wei, Y. (2021). A spherical harmonic Martian crustal magnetic field model combining data sets of MAVEN and MGS. Earth and Space Science, 8(10), e2021EA001860. https://doi.org/10.1029/2021EA001860
3. P. K. Seidelmann (Chair), V. K. Abalakin, M. Bursa, et al (2002). Report of the IAU/IAG Working Group on Cartographic Coordinates and Rotational Elements of the Planets and Satellites: 2000. Celestial Mechanics and Dynamical Astronomy, 82, 83–111. https://doi.org/10.1023/A:1013939327465
4. Xu, S., Weber, T., Mitchell, D. L., Brain, D. A., Mazelle, C., DiBraccio, G. A., & Espley, J. (2019). A Technique to Infer Magnetic Topology at Mars and Its

Application to the Terminator Region. Journal of Geophysical Research: Space Physics, 124(3), 1823-1842. https://doi.org/10.1029/2018ja026366

5. Büchner, J., & Zelenyi, L. M. (1989). Regular and chaotic charged particle motion in magnetotail like field reversals: 1. Basic theory of trapped motion. Journal of Geophysical Research, 94(A9). https://doi.org/10.1029/JA094iA09p11821
